# The pressure field as a methodology for fluid management and red cell preservation during cardiac surgery

**DOI:** 10.1186/s13019-023-02134-3

**Published:** 2023-01-18

**Authors:** Stephen F. Woodford, Mark Butlin, Bai Wei, Wei Chao, Alberto Avolio

**Affiliations:** 1 Department of Anesthesia, Austin Health, Heidelberg, Australia; 2 Department of Anesthesia and Intensive Care, Brisbane Waters Private Hospital, 21 Vidler Ave., Woy Woy, Australia; 3 Department of Anesthesia and Intensive Care, Gosford Private Hospital, Burrabil Ave, Gosford, Australia; 4 Faculty of Medicine, Health and Human Sciences, Macquarie Medical School, Macquarie University, Sydney, Australia; 5 Beijing Technology and Business University, Beijing, China; 6 Shanghai Tongji University, Shanghai, China

**Keywords:** Hemodynamics, Fluid therapy, Patient blood management, Transfusion

## Abstract

**Purpose:**

Anemia and red cell transfusion contribute to morbidity and mortality of surgery. The concept of patient blood management to mitigate preoperative anemia, optimize coagulation, conserve red cells intraoperatively and accept lower post-operative transfusion thresholds has recently gained widespread acceptance across a range of surgical disciplines. Fluid administration is likely to contribute significantly to perioperative anemia and red-cell transfusion requirements, yet a robust basis for managing fluid administration in this context has not been articulated. There is an urgent need for this.

**Methods:**

We developed ‘the pressure field method’ as a novel approach to guiding the administration of fluid and drugs to optimize tissue perfusion. The pressure field method was used for the intraoperative management of 67 patients undergoing semi-elective cardiac surgery. We compared intraoperative anemia and transfusion requirements in this cohort with a conventional group of 413 patients undergoing cardiac surgery.

**Results:**

In the pressure field group, no patients required transfusion whereas in the conventional group, 16% required transfusion during bypass and these patients received an average of 2.4 units of packed red cells (P < 0.0001). The average decrease in hemoglobin in the pressure field group was only 13 g/L, whereas in the conventional group it was 52 g/L (P < 0.0001). 80% of the pressure field group received no intravenous fluid during cardiac surgery, and the average intraoperative fluid load was 115 mL.

**Conclusion:**

The pressure field method appears to reduce transfusion requirements due to decreased intraoperative fluid loading.

**Supplementary Information:**

The online version contains supplementary material available at 10.1186/s13019-023-02134-3.

## Introduction

Anemia is an independent risk factor for surgical morbidity and mortality [1, 2, 3], but red cell transfusion is not the answer. Red cell transfusion causes an increase in early [4] and late [5, 6] morbidity and mortality: it is immunomodulatory [7] and increases surgical reintervention [8], postoperative infection rates [9, 10] and cancer-related mortality after cystectomy [11], and after surgery for colorectal cancer [12] and gastric cancer [13]. Further, the morbidity associated with the combination of anemia and red cell transfusion is greater than their additive effects [14]. If anemia and red cell transfusion both increase morbidity, then the argument for avoiding both is crystal clear. Since Isbister [15] proposed the concept of patient blood management (PBM), this multi-modal approach to mitigate preoperative anemia, optimize coagulation, preserve red cells during surgery and tolerate lower transfusion thresholds following surgery has gained broad acceptance [16, 17].

PBM is particularly relevant to cardiac surgery, as more than 50% of cardiac surgical patients receive a transfusion [6, 7]. Schwann et al. [5] argued that transfusion causes a doubling of all-cause mortality at 5-years after coronary artery bypass grafting, and that transfusion is an independent predictor [18] of early coronary occlusion. Other effects of red cell transfusion after cardiac surgery include atrial fibrillation [19] and sepsis [9]. To address this issue, in 2007 the Society of Thoracic Surgeons Blood Conservation Guideline Task Force [20] examined the evidence base for each component of PBM in cardiac surgery, with the guidelines updated in 2021 [16]. The guidelines recommend meticulous attention to hemostasis, the use of antifibrinolytics to reduce perioperative bleeding, acute normovolemic hemodilution with post-bypass reinfusion, the use of retrograde autologous priming of the bypass circuit, and routine use of red cell scavenging and reinfusion following centrifugation (but not the direct reinfusion of shed mediastinal blood). The benefit of this systematic approach has been validated [21]. The guidelines provide an important synopsis of the problem and helpful guidance, while assuming that the principal drivers of perioperative transfusion are inadequate erythropoiesis and intraoperative red cell loss.

Fluid administration is one of the key clinical interventions in cardiac surgery, and fluid is typically administered in litres: Shaw [22] claimed that crystalloid administration is invariable in cardiac surgery, Mythen [23] estimated the volume of intraoperative fluid loading during cardiac surgery at 3–5 L, and Vretzakis [24] reported an average intraoperative fluid load of 3 L during cardiac surgery. The role of fluid administration in causing hemodilution and increasing red cell transfusion requirements has not been extensively studied, but administration of fluid apart from blood necessarily causes a dilutional reduction in hemoglobin. The clinical conundrum is how to titrate fluid to maintain perfusion while avoiding the morbidity and mortality of hemodilution and red cell transfusion. Given its multifactorial role in tissue perfusion and oxygen delivery, patient fluid management should form an important component of the PBM bundle.

We developed the pressure field method to assist in managing perfusion. The method combines continuous hemodynamic measurement with real-time computer-based visualization of blood pressure regulation patterns (the ‘pressure field’ visualization). The pressure field visualization enables the detection of small directional changes in the beat-to-beat ventricular and vascular contributions to the generation of blood pressure, and enables small adjustments to fluid, inotropes, and vasoactive drugs to be made at the highest available frequency of measurement. We observed that the real-time visualization of the pressure field was impacting clinical management of fluid and pressor administration [25], and undertook an ambidirectional observational study of fluid administration and transfusion rates in semi-elective cardiac surgery patients.

## Methods

### Study design and oversight

An ambidirectional study of 480 semi-elective cardiac surgery patients at two community hospitals was undertaken. The study was approved by the Macquarie University Human Research Ethics Committee in Australia (Ethics Ref 5201200007), Hunter New England Human Research Ethics Committee (Ref 13/10/16/4.03), and by the Medical Advisory Committees of the two community hospitals. Patients with a logistic Euroscore [26] predicted mortality below 10% undergoing on-pump or off-pump cardiac surgery were included in the study. Data was prospectively collected with patient consent for the 67 patients managed using the pressure field between 2014 and 2017. For the conventional group, data was retrospectively extracted from a database of patients undergoing cardiac surgery between 2004 and 2012. Data on age, sex, surgery type, pre- and post-operative blood hemoglobin concentrations, and units of packed red blood cells given during bypass was collected for all patients. Intraoperative hemodynamic and intravenous fluid data was collected for the pressure field group; this data was not available for the conventional group.

### Transfusion principles

For all patients, arterial blood gas measurement was performed prior to surgery, during surgery and cardiopulmonary bypass, and on completion of surgery. In all patients, allogeneic donor blood was transfused if hematocrit decreased below 20%, or if there was hemodynamic instability with a downward-trending blood hemoglobin.

### Measurement and management of perfusion using fluid and vasoactive drugs

All patients were monitored with a radial intra-arterial pressure line, a central venous line, and a pulmonary artery catheter (Edwards Lifesciences, CA, US). In most of the conventional group and all the pressure field group, patients were also monitored using a FloTrac arterial transducer, a TruWave central venous pressure transducer and the EV1000 hemodynamic monitoring platform (Edwards Lifesciences, CA, US).

In the pressure field group, following insertion of lines and zeroing of all transducers, a continuous stream of pressure and stroke volume (SV) data was transmitted from the EV1000 monitoring platform via a serial-to-USB cable to an investigational software tool which displayed a ‘pressure field’. The mean arterio-venous pressure gradient (that is, [MAP–CVP]) of a specific cardiac cycle can be defined in terms of the SV contributed by the heart and the systemic elastance (Es) contributed by the vasculature such that:

1 $$\left[ {MAP - CVP} \right] = SV \times Es$$

where MAP is mean arterial pressure, CVP is central venous pressure, SV is stroke volume and Es = systemic elastance.

**Fig. 1:**
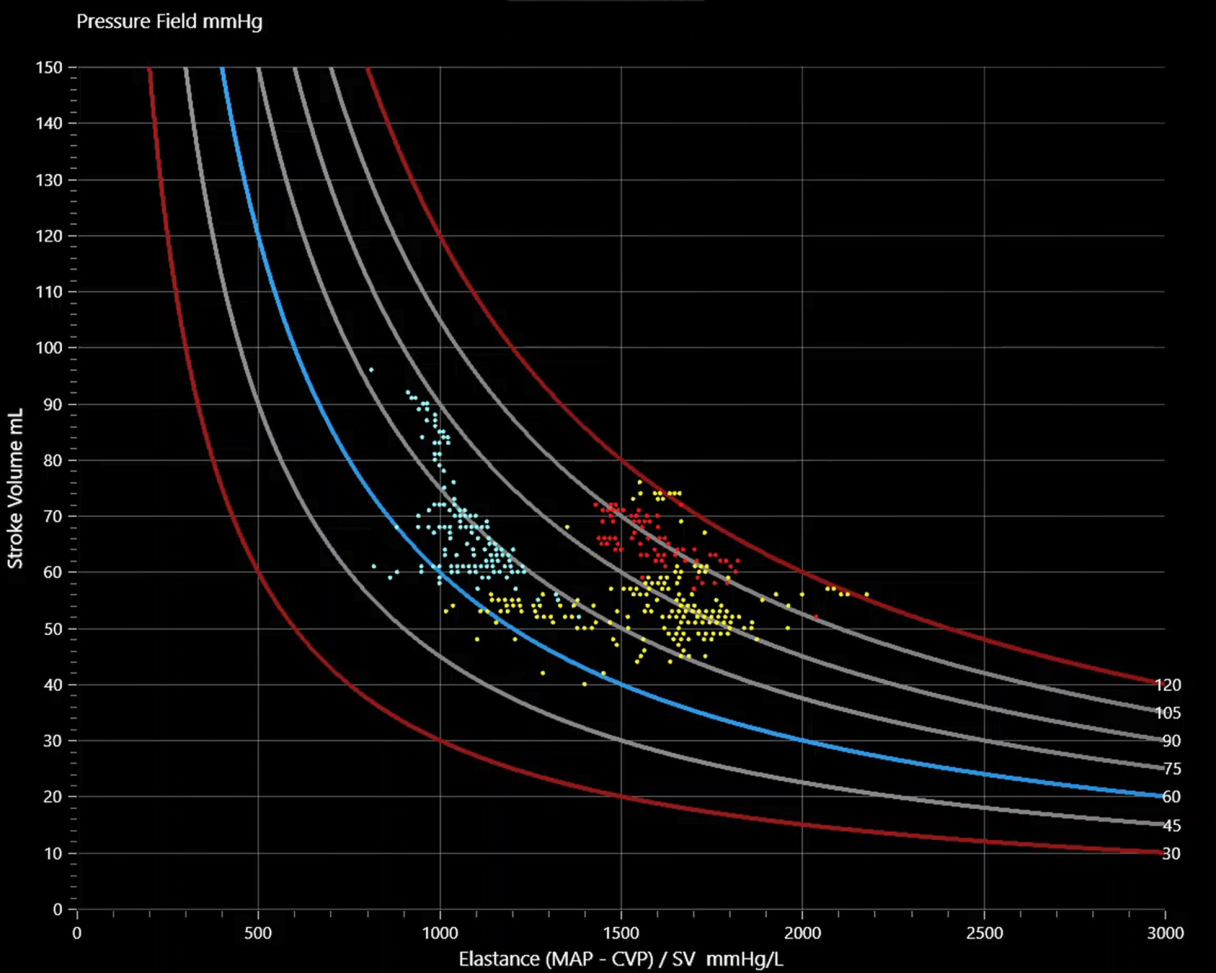
The pressure field visualization. Each dot represents a specific SV, Es, and a resulting MAVP gradient which is indicated by curved lines. Color is used to designate clinically significant events, interventions, or time intervals. In this example of coronary artery graft surgery, red dots indicate the pre-induction period, yellow dots indicate the pre-bypass period and blue dots represent the post-bypass period. The curved blue line indicates an arterio-venous pressure difference of 60 mmHg

The high-frequency plotting of values according to Eq. (1) defines a ‘pressure field’. Es was calculated at the frequency at which MAP, CVP, and SV were measured in the intact circulation by the EV1000 monitoring platform, that is every 20 s. The three parameters of [MAP–CVP], SV, and Es were displayed in a 2D visualization (see Fig. 1). This visualization provides a real-time graphical view, updated every 20 s, of how ventricular-vascular interaction produces a particular pressure.

The ‘pressure field’ defined over a short interval before induction of anaesthesia generated a patient-specific template to guide the administration of fluid, pressors and inotropes during the pre-bypass period and after weaning from bypass. Vertical movements in SV may signify changes in cardiac volume (preload) or contractility, with the relative importance of each differentiated by the response to interventions; Hartmann’s solution was administered in boluses of 1 mL/kg to address issues of preload with the roller clamp otherwise kept closed, and norepinephrine was administered to address issues of contractility. Horizontal movements in Es signify changes in vasomotor tone or intra-vascular volume, with norepinephrine (NE) and low-dose glyceryl trinitrate (GTN) commenced at induction and then titrated to minimise movement in Es. Norepinephrine at 4 mcg/min was commenced 10 min before induction to prime the central venous line and the dose was then varied according to changes in the pressure field (typically between zero and 10 mcg/min), and GTN was given in a concentration of 1 mg/mL at an infusion rate of 1 mL/h throughout surgery.

All patients had two large-bore peripheral venous cannulas. In the conventional group, patients had a freely running infusion of Hartmann’s solution, and in the pressure field group both lines were clamped. Management of perfusion was otherwise according to anesthesiologist preference with NE, GTN, and dobutamine or milrinone administered as required.

In both the conventional group and the pressure field group, pressure was deliberately lowered for harvesting of venous and arterial conduits where this was requested by the surgeon, and before going onto bypass. After weaning from bypass, perfusion pressure was maintained above 65 mmHg. The conduct and management of cardiopulmonary bypass in both groups was performed by a board certified perfusionist.

The principal difference of the pressure field from the conventional group was the titration of fluid and drugs based on continuous visualization of a ‘pressure field’ (see Additional file 1).

### Additional PBM interventions

Additional PBM techniques were employed. In both the conventional and pressure field groups, subjects were administered antifibrinolytics (viz. tranexamic acid) at induction and during surgery, there was careful attention to hemostasis, and the cardiopulmonary bypass circuit was primed with Ringer’s solution. In the conventional group, pump blood was directly reinfused at the completion of bypass. In the pressure field group, pump blood was directly reinfused at the completion of bypass in 48 patients; in 17 patients a cell-saver was used to scavenge blood in the surgical field, and on completion of bypass the scavenged and pump blood was spun down and reinfused as a red cell concentrate. Acute normovolemic hemodilution was not used in either group.

### Outcomes and statistical analysis

The primary outcomes of the study were the change from pre-operative to completion-of-surgery blood hemoglobin, and the quantity of packed red blood cells transfused during surgery. Intravenous fluid load for the pressure field group was also examined.

Numerical variables for the conventional group and the pressure field group were compared by Welch two sample t-test, or where non-normally distributed a Wilcoxon rank sum test. Categorical variables were compared by Chi-squared test. Stepwise multiple linear regression was performed to find the predictors of blood hemoglobin concentration change, with the inputs of the statistical model being age, sex, fluid management technique (conventional or pressure field), pre-surgical blood hemoglobin concentration, and surgery type. The relationship between administered fluid where known, including units of blood, was investigated in relation to changes in hemoglobin concentration by linear regression. Analysis was conducted in R (version 4.1.1).

## Results

### Patients

Data sets were collected from and collated for a total of 480 patients. 413 patient data sets were collated for conventional management and 67 patient data sets were collated for pressure field management. There was no difference in age between the two groups (p = 0.589, Table 1) though there were more females in the conventional group (26% vs. 13%, p = 0.026, Table 1).

**Table 1:** Group comparison

	Conventional (n = 413)	pressure field (n = 67)	p
Age (years)	69 ± 10	68 ± 8	0.569
Sex (% female)	26	13	**0.027**
Pre-operative [Hb] (g/L)	139 ± 15	134 ± 16	**0.035**
Post-operative [Hb] (g/L)	87 ± 12	121 ± 24	**< 0.0001**
Change in [Hb] (g/L)	− 52 ± 14	− 13 ± 7	**< 0.0001**

Despite pre-operative blood haemoglobin concentration being lower in the pressure field group than the conventional group, the post-operative blood haemoglobin concentration is higher in the pressure field group than the conventional group. p-value by t-test, with p-values < 0.05 indicated in bold type

### Pre- and post-operative blood hemoglobin concentration

**Fig. 2:**
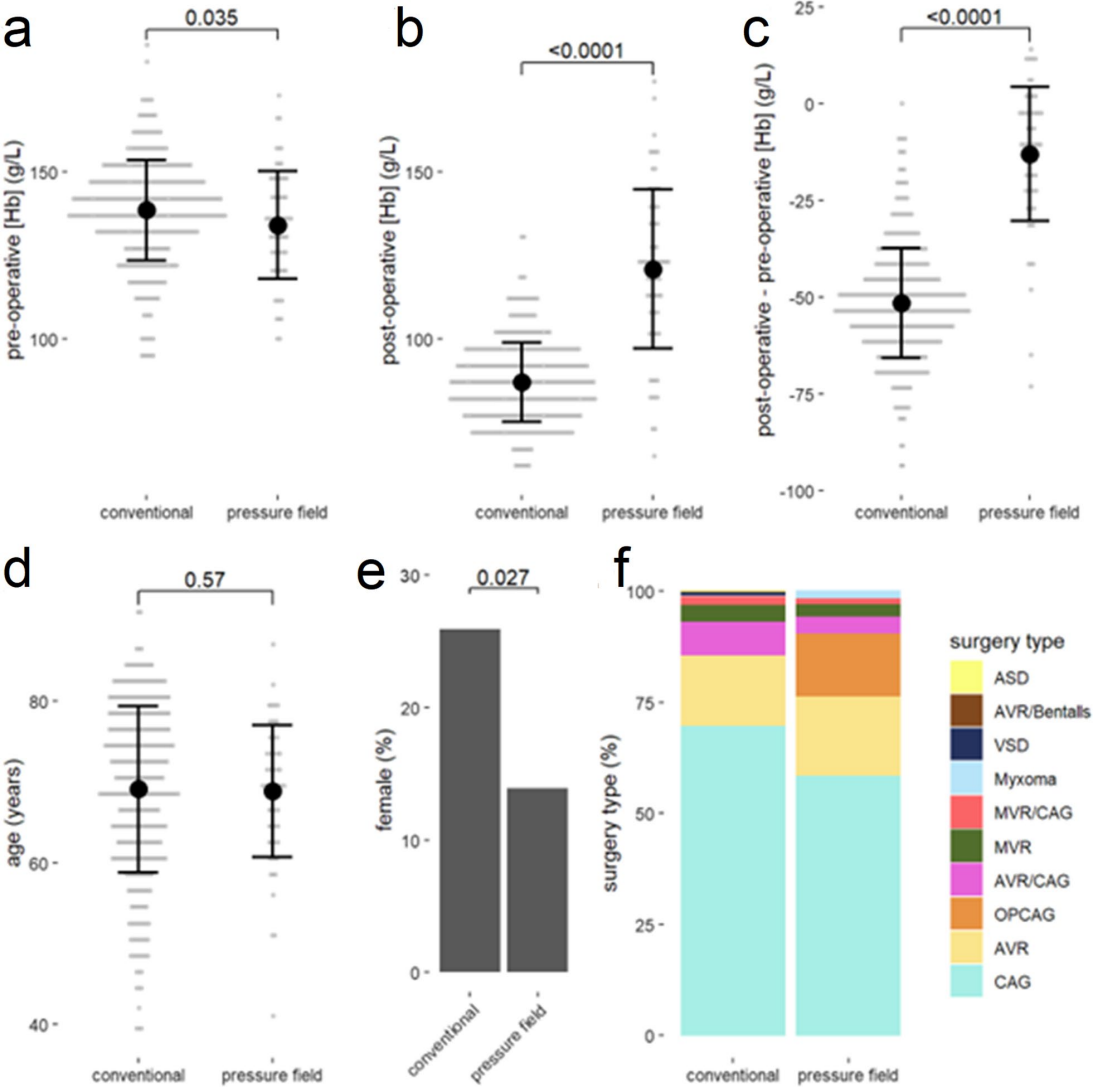
**a** The pre-operative blood haemoglobin concentration, **b** post-operative blood haemoglobin concentration and **c** pre- to post-operative difference in blood haemoglobin concentration were different between conventional and pressure field perfusion management. **d** There was no difference in age between the two groups. **e** There was a difference in sex between the two groups. **f** There was a difference in the proportional magnitude of types of surgery between the two groups (p < 0.001). Grey dots show raw data. Black dot shows mean with ± 1 standard deviation bar displayed

**Fig. 3:**
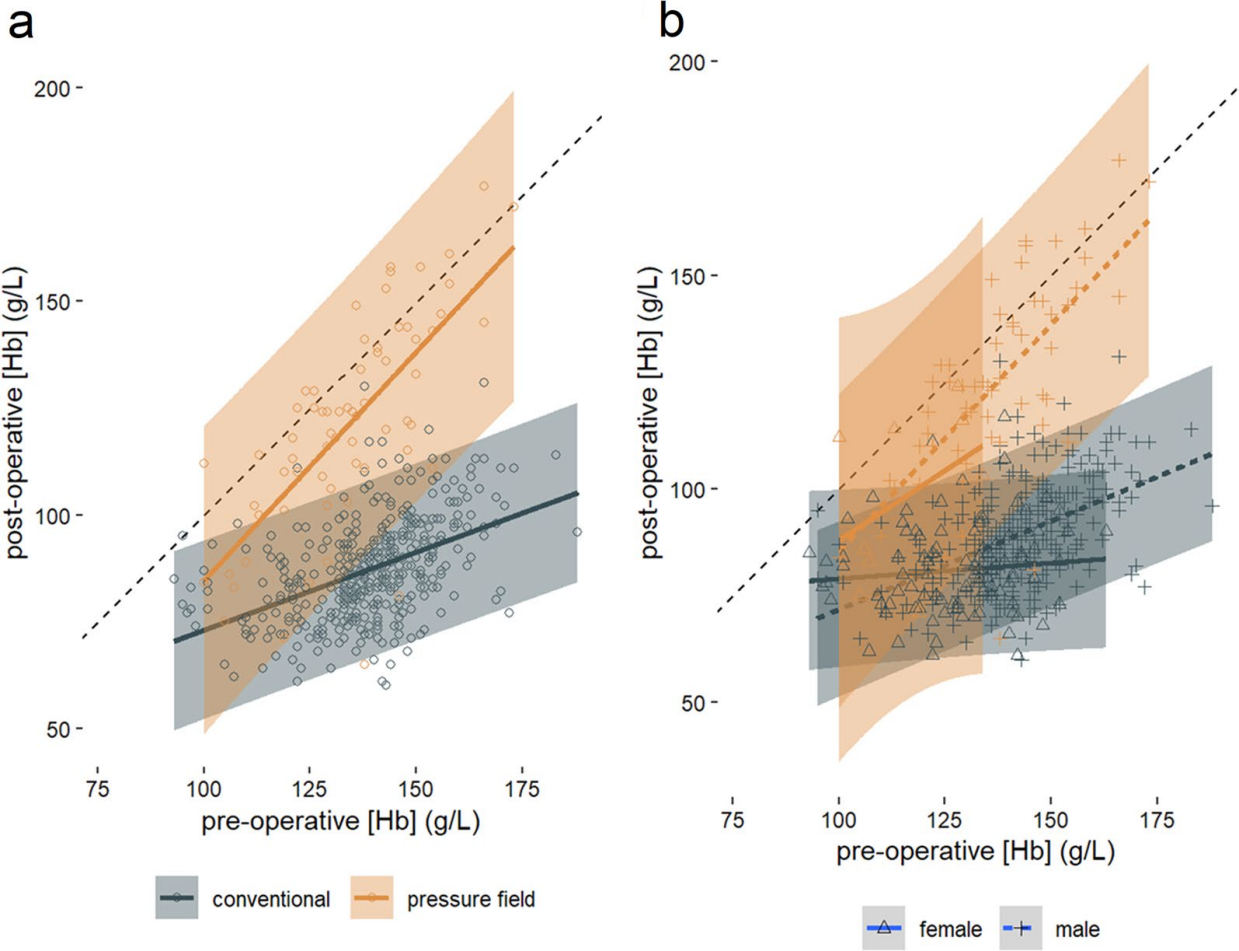
The pre-operative and post-operative blood haemoglobin concentration for conventional perfusion management and perfusion management using the pressure field. The dashed line is the line of identity where post-operative and pre-operative haemoglobin are equal. Shaded region gives the 95% confidence interval. **a** The slope (p < 0.001) and intercept (p < 0.001) of the two groups was different. That is, the pressure field group was closer than the conventional group to the line of unity where post-operative haemoglobin is equal to pre-operative haemoglobin. **b** Plot showing the significant (p < 0.001) pre-operative/post-operative blood haemoglobin concentration interaction with sex. Within each sex the difference in conventional or pressure field approach remains. Regression coefficients are provided in Table 2

There was a smaller decrease in blood hemoglobin concentration through surgery in the pressure field group than in the conventional group (p < 0.0001, Table 1, Fig. 2b). Pre-operative hemoglobin concentration was lower in the pressure field group than in the conventional group (p = 0.035, Table 1, Fig. 2c) and yet post-operative hemoglobin concentration was higher in the pressure field group than the conventional group (p < 0.0001, Table 1, Fig. 2d). Pre- and post-operative hemoglobin concentration was correlated, with post-operative hemoglobin concentration being closer to pre-operative hemoglobin concentration in the pressure field group (Table 2, Fig. 3a).

**Table 2:** Regression coefficients of pre-operative and post-operative hemoglobin concentration, as visualised in Fig. 2

Method	Slope	intercept	R^2^	P
Conventional	0.36 ± 0.03	36 ± 5	0.212	**< 0.0001**
Pressure field	1.11 ± 0.13	− 22 ± 18	0.491	**< 0.0001**
Conventional—female	0.07 ± 0.07	72 ± 9	0.002	0.291
Pressure field—female	0.65 ± 0.55	23 ± 64	0.047	0.275
Conventional—male	0.41 ± 0.04	30 ± 6	0.234	**< 0.0001**
Pressure field—male	1.06 ± 0.16	− 21 ± 21	0.444	**< 0.0001**

A slope of ‘1’ indicates no change in hemoglobin between the beginning and completion of surgery; a zero slope indicates a constant final hemoglobin, irrespective of the initial value. Correlation is shown for the combined male and female groups, and the groups are then classified by both method and sex. P-values < 0.05 are indicated in bold type

The interaction with sex was visually examined (Table 2, Fig. 3b). This was backed by a stepwise multiple linear regression model that showed a dependence on both method of management (standardised ß = 0.58 ± 0.03, p < 0.0001) and sex (standardised ß = -0.10 ± 0.03, p = 0.0008) but with no significant interaction between management method and sex (p = 0.106).

Surgery type differed between the two groups (Fig. 2a) and certain surgery types were predictive of the pre- to post-surgical change in hemoglobin concentration (Table 3). There was an interaction of the surgery type with method of perfusion management for mitral valve replacement, aortic valve replacement, and atrial septal defect. However, with inclusion of surgery type in the stepwise linear regression model, management type (conventional or pressure field) remained the strongest predictor of the change in hemoglobin concentration through the surgical procedure (Table 3).

**Table 3:** Resulting model from stepwise multiple regression with pre- to post-operative change in blood haemoglobin concentration

Term	ß	Standardised ß	p
Method (conventional/pressure field)	41 ± 2	0.58 ± 0.03	**< 0.0001**
Initial [Hb]	− 0.57 ± 0.04	− 0.44 ± 0.03	**< 0.0001**
CAG	− 6.1 ± 1.9	− 0.13 ± 0.04	**0.0012**
Method * MVR	− 29 ± 7	− 0.12 ± 0.03	**< 0.0001**
Method * AVR	− 16 ± 4	− 0.12 ± 0.03	**< 0.0001**
Female	− 0.04 ± 0.01	− 0.10 ± 0.03	**0.0008**
Myxoma	− 18 ± 9	− 0.06 ± 0.03	**0.0142**
AVR	− 0.13 ± 1.85	− 0.05 ± 0.04	0.1957
MVR	2.6 ± 2.8	− 0.02 ± 0.03	0.5592

The method (conventional/pressure field) had the largest impact with a standardised β of 0.58 ± 0.03. Table ordered from largest to smallest absolute standardised β value. P-values < 0.05 are indicated in bold type

### Transfusion of packed red blood cells

**Fig. 4:**
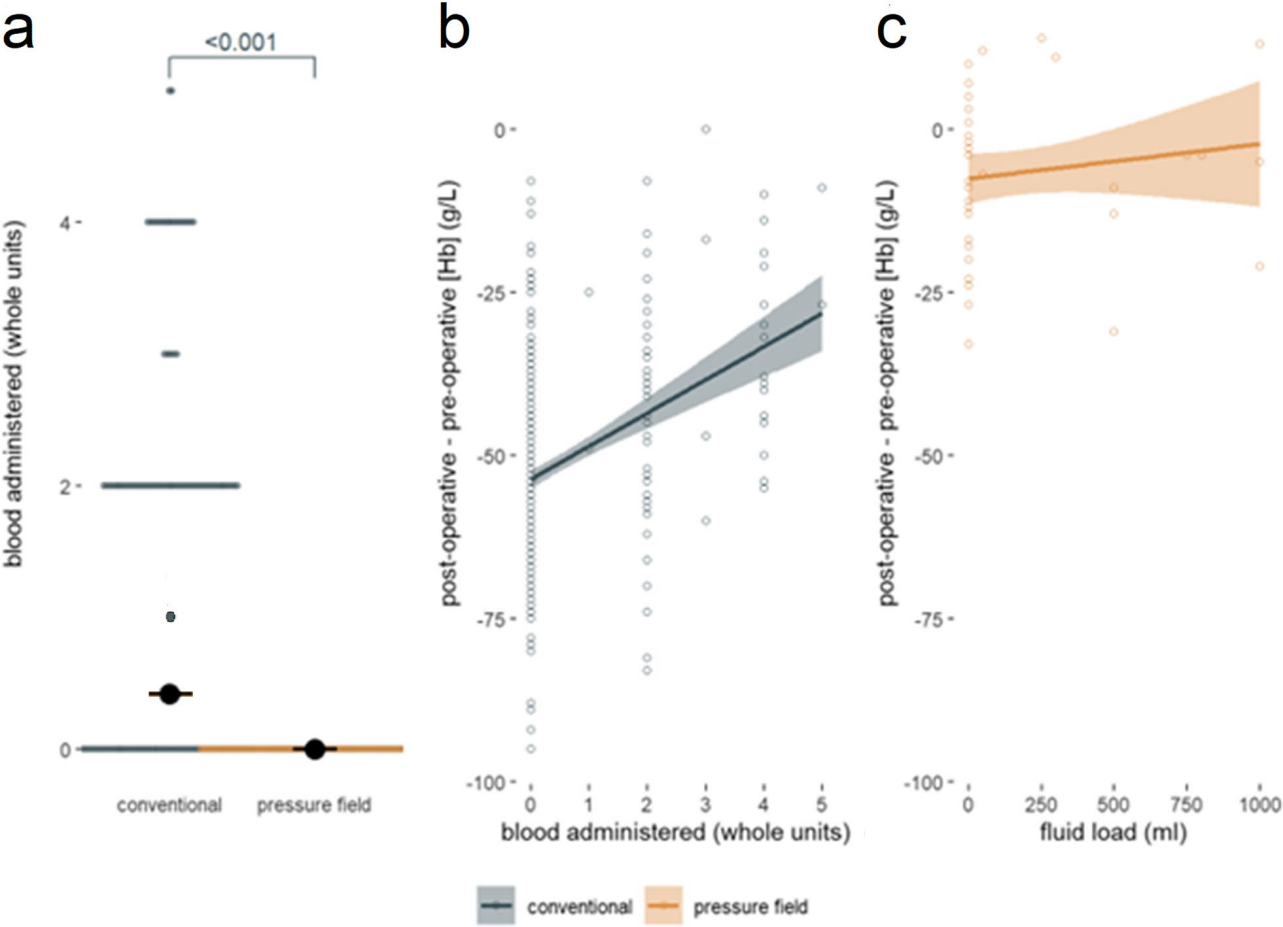
**a** An average of 0.4 units of whole blood was administered in the conventional group per patient. No blood was administered in the pressure field group. **b** Change in haemoglobin concentration was positively correlated with blood transfused in the conventional treatment group (∆[Hb] = 5.1 × units of blood-54 g/L, R^2^ = 0.134, p < 0.001). **c** There was no correlation between intravenous fluid administered in the pressure field management group and change in haemoglobin concentration. Fluid load data unavailable for the conventional treatment group

The lower post-operative hemoglobin concentration in the conventional group was despite, on average, 0.4 units of blood administered per patient (median value nil units, range 0–6 units) whilst no blood was administered in the pressure field group (p < 0.001, Fig. 4a). In the conventional group, 16% were transfused an average of 2.4 units of packed red cells; and the greater the amount of blood administered, the smaller the change in hemoglobin concentration through surgery (Fig. 4b).

### Intravenous fluid administration

The volume of intravenous fluid administration was not available in the conventional group but was the likely driver of the decreased hemoglobin concentration in this group. The mean volume of intravenous fluid administered per patient in the pressure field group was 115 mL with a median infusion volume of zero. 54 patients in the pressure field group received no fluid, and the remaining 13 patients received 50 mL to 1 L, or an average volume of 592 mL. There was no correlation between fluid administration in the pressure field management group and change in hemoglobin concentration (Fig. 4c).

## Discussion

### Key findings

The pressure field provides a methodology for perfusion management, and its application leads to different treatment choices [25]. The pressure field group experienced a much lower average fall in hemoglobin of 13 g/L compared with 52 g/L in the conventional group. This was despite 16% of the conventional group receiving an intraoperative transfusion, and transfused patients experiencing a lower observed drop in blood hemoglobin than patients in the conventional group who did not receive a transfusion (Fig. 4b). In contrast, no patients in the pressure field group received a blood transfusion. The average amount of fluid given in the pressure field patients was 115 mL, or less than one standard drink; 54 of the 67 patients in the pressure field group received no fluid at all. The evidence supports the conclusion that patients managed with the pressure field received less fluid—although restriction of fluid was not the goal of the pressure field method per se—and that this resulted in better preservation of blood hemoglobin concentration and decreased transfusion requirements.

### Implications

The pressure field method is a significant departure from the traditional approach to perfusion management. Rather than protecting a generic pressure target or generic cardiac output and systemic vascular resistance values, the real-time display of the pressure field enables the anesthesiologist to deconstruct perfusion pressure quickly and visually into ventricular and vascular components, with the goal of replicating a patient’s pre-induction perfusion patterns during surgery. Horizontal movements in Es are the result of changes in intravascular volume or vasomotor tone and treated with vasopressors or vasodilators. Vertical movements in SV may result from changes in cardiac volume (preload) or contractility and can be distinguished by administering a small dose of fluid (typically 1 mL/kg) or a vasoactive drug and observing the SV response; fluid is used to address issues of preload, and inotropes to address issues of contractility. The pressure field therefore provides a method of differentiating changes in preload, afterload and contractility and provides a basis for when to treat low pressure with fluid, inotropes, or pressors, or some combination of all three—and in what doses. Diagnosis is still an empiric process of trial and error but with smaller interventions and fewer and smaller errors. In addition, the pressure field enables individualized management: the patient’s own physiology and the unique patterns of displacement from this physiology direct clinical decision making. Since the pressure field provides a continuous measure of afterload, it is of particular value in old age, where vascular stiffness is increased.

Vretzakis et al. [24] were able to reduce transfusion rates in a comparable cardiac surgical population from 67 to 17% by applying a restrictive fluid strategy and routinely reinfusing salvaged washed red cells. With a novel method of perfusion management, the pressure field eliminated the need for transfusion and all but eliminated the need for intravenous fluid during elective cardiac surgery.

The pressure field is a refinement and application of the work of Sunagawa [27] on ventricular preload and afterload in an isolated canine ventricle model, which itself was an extension of Otto Frank's work [28, 29]. Although Sunagawa is primarily cited for his work on ventricular-arterial coupling, his insight that ventricular afterload is more accurately represented as an elastance [30] than as Guyton’s systemic vascular resistance [31] is the key insight behind the pressure field model. The relatively recent improvements in both the accuracy and high frequency of estimation of SV in the intact circulation have created the opportunity to apply Sunagawa’s insight into afterload to anesthesia, with CVP used as the measure of downstream pressure (Pd); Sunagawa took the view that Pd varies with and is greater than CVP, however CVP remains the best available estimate of Pd in the intact circulation. It is important to note that Eq. (1) is mathematically similar to Guyton’s pressure equation, but with time removed from both CO and SVR. The pressure field provides a visual representation of continuous ventriculo-arterial coupling.

Despite randomized trials, meta-analyses and the recommendations of professional societies on the principles of PBM, effective therapies to prevent low intraoperative haemoglobin concentration have not been well validated [32] and evidence for a mortality benefit from PBM protocols is still lacking. The pressure field approach to PFM may prevent low intraoperative haemoglobin concentrations and therefore complement existing PBM techniques.

### Strengths and limitations

The strengths of this study include the large number of patients in the pressure field group, and precise documenting of hemodynamic parameters as well as red cell transfusions and fluid volumes for this group.

Surgery in the conventional group occurred in years prior to surgery in the pressure field group, however the guiding principles of perioperative red cell transfusion did not change during the study period and this does not detract from the key observation that transfusion rates and hemodilution in the pressure field group were very low.

All the conventional group were on-pump while 59 of the 67 patients in the pressure field group were on-pump, and crystalloid priming volumes for the extracorporeal circuit were not recorded for either group. The dilutional effect of the crystalloid prime does not explain hemoglobin and transfusion differences between the conventional and pressure field groups, given the extent of the differences and small proportion of off-pump cases in the pressure field group. Although a small but significant difference in transfusion requirements between on and off-pump cases has been documented by others [33, 34, 35], none of the pressure field patients received a blood transfusion.

Red cell scavenging and reinfusion was used in 17 of the pressure field group; the impact of this is not able to be separately quantified but is expected to be small. Data on post-bypass transfusion of allogeneic donor blood was not available for the conventional group, whereas no patient in the pressure field group received any blood.

There were comparatively fewer females than males (Table 2), possibly indicating that the study was underpowered for the female sex. However there was the statistical power for sex to show an effect on change in hemoglobin through surgery (Table 3), which may have resulted from the smaller average body mass of women and commensurately greater impact of priming volumes in the extra-corporeal circuit on the dilution of hemoglobin.

Statistical inference was used to correct for confounders and leads to the conclusion that perfusion management was the main contributor to the differences in blood hemoglobin concentrations and transfusion requirements between the conventional and pressure field groups.

## Conclusion

This study of patients undergoing cardiac surgery indicates that the pressure field-guided management of 67 patients led to better preservation of blood hemoglobin and a lower red cell transfusion rate due to decreased perioperative fluid loading when compared with a historic control group of 413 patients. Donor blood transfusion was not merely reduced, but eliminated in the pressure field group, yet with a better-preserved hemoglobin in this group at the end of surgery. The significant differences in hemoglobin and transfusion rates between the pressure field and conventional groups warrant the design of a prospective randomised trial in patients undergoing cardiac surgery. The pressure field method may be a useful addition to the current patient blood management bundle.

## Supplementary Information

**Additional file 1**: The supplementary video file describes the principles of the pressure field method, with particular reference to application of the method during coronary artery surgery.

## Data Availability

The datasets used and/or analysed during the current study are available from the corresponding author on reasonable request.
